# Physics-Informed Directed Graph Network-Based Temperature Forecasting Model

**DOI:** 10.3390/s25175295

**Published:** 2025-08-26

**Authors:** Jinjing Cai, Binting Su, Shuping Chen, He Fang

**Affiliations:** 1 Fujian Province Warning Information Release Center, FuZhou 350000, China; jinjincai1995@hotmail.com; 2 College of Computer and Cyber Security, Fujian Normal University, FuZhou 350117, China; silence.11.yiou@gmail.com (S.C.);

**Keywords:** temperature prediction model, physics-informed model, directed graph network

## Abstract

Recently, there has been a dramatic rise in the demand for accurate temperature forecasts. However, challenges arise from modeling and fusing complex spatial and temporal features in temperature data. In this study, we propose a physics-informed directed-graph-based temperature prediction model to mitigate the challenges of purely data-driven prediction algorithms. Firstly, a directed graph design module was designed and then used to construct an asymmetric adjacency matrix based on the locations of temperature-monitoring stations. This module can capture the asymmetric relations between temperature data at different stations. Then, the directed adjacency matrix was incorporated into the graph attention module and the graph-gating module to extract the spatial and temporal features of the temperature data, and a fusion module was designed to integrate the spatial–temporal features and the directed graph adjacency matrix to provide better temperature prediction performance. Numerical simulations based on a real-world dataset collected in southern China demonstrate that our proposed physics-informed temperature prediction model can deliver superior prediction performance with a mean absolute error of less than 0.75 °C.

## 1. Introduction

Accurate weather forecasting is important for economic development and industrial production in modern society. This includes outdoor work, factory production, agricultural production, green power generation, the prevention of natural disasters, and the creation of comfortable living and working environments for humans [1]. Sustained increases in long-term average temperatures will lead to global warming and the emission of greenhouse gases [2]. These gases can also cause significant temperature variations over short periods, resulting in extreme weather events such as heatwaves and severe droughts [3,4]. Therefore, an increasing number of researchers are focusing on high-accuracy air temperature prediction models, and many studies have explored the potential of advanced techniques to improve traditional air temperature prediction models.

Numerical temperature prediction models utilize mathematical models of the atmosphere, land, and oceans to predict temperatures based on current weather conditions [5]. These mathematical models are developed based on physical principles and can be used to generate short-term weather forecasts or long-term temperature predictions. These models are used by weather centers around the world to effectively simulate global and regional temperature dynamics. Although numerical prediction models are reliable, they require significant computational resources. Moreover, inaccuracies in numerical temperature forecasting models remain inevitable due to the flaws in the initial field of the numerical model, shortcomings in model physics, and the chaotic nature of weather fluctuations [6].

Statistical modeling is widely used in time series prediction and temperature forecasting. For example, the Autoregressive Integrated Moving Average (ARIMA) model [7] and the Seasonal ARIMA model [8] were developed by utilizing the linear relationship between temperature measurement data. These data are assumed to follow a statistical distribution. To improve the accuracy of temperature prediction, nonlinear relationships of measured temperature data have been investigated. A nonlinear autoregressive exogenous (NARX) model [9] has been developed for predicting the temperature of a solar greenhouse. A support vector regression algorithm [10] has been designed to predict the temperature over a 24 h period, by considering relative humidity, air pressure, and synoptic situations.

The successful application of neural networks in areas such as image recognition [11,12], scene understanding [13], and large-language modeling [14,15] has also facilitated research into their use in weather prediction. A convolution-neural-network-based CU-net [16] was employed to correct the bias of numerical weather predictions to improve the acccuracy of severe weather warnings. A data-driven neural network method [17] was designed to incorporate nonlinear relationships between arbitrary variables and temperature distributions without prespecified link functions. It provides insight into the impact of meteorological variables on temperature predictions and is more affordable in terms of computational cost. Recurrent neural networks (RNNs) are the most frequently used neural network design for sequence prediction issues. An RNN-based method [18] was designed to generate synthetic, localized weather data that are significantly more accurate and representative of local conditions than standard weather files. This method can be used to modify generic weather files to accurately reflect local conditions or generate localized data over a longer time span when only a subset of the data are available or have been collected. A long short-term memory (LSTM)-based deep-learning model [19] was used for sea surface temperature prediction. It was further applied to the issue of abnormally high water temperatures. A monthly climate prediction model was designed by combining CNN and LSTM structures [20]. This model uses 72 years of monthly average atmospheric temperatures as training data and can predict temperatures one month in advance. A 2D-convolutional multivariate LSTM model [21] was developed for short-term temperature prediction to utilize data collected from different weather stations in Germany. The nonlinear relationships between temperature, air pressure, and dew point, as well as wind speed, were integrated into an CNN-LSTM-based model [22] for predicting hourly air temperature. The CNN reduces the dimensionality of the time series data, while the LSTM captures its long-term memory. A separable CNN-LSTM model [23] was used to predict lake water temperature by using only temperature as the model input; air temperature, solar irradiance, and wind speed are measured, and lake water temperature is measured hourly at 12 different depths. A deep learning-based proxy model employing a generative adversarial network [24] has been proposed for prediction of temperature evolution during CO_2_ circulation, where permeability fields and time are used as inputs to estimate temperature distributions. A stacked ensemble of deep learning models [25] has been designed for temperature prediction, which shows a better spatial distribution for the temperature prediction of the sea surface.

The aforementioned methods treat each sensor as an individual entity, ignoring the topology and relationships of the temperature monitoring sensor network, as well as its connectivity. Hence, graph neural networks have been studied in the field of temperature predictions. A time series graph network [26] has been proposed to jointly capture the spatial corrections of sensor measurements by graph structure, where a long short-term memory module has been designed to aggregate the temporal features of time series data. To improve sea surface temperature prediction performance, a three-dimensional (3D) dynamic spatio-temporal graph neural network [27] was proposed, employing the full range of dynamic spatial properties of the ocean temperature field based on fine-grained modeling of spatial correlations. A global spatio-temporal graph attention network [28] was created to predict sea surface temperatures, where the global dynamic spatial correlations of sensors can be captured by a global graph attention module, and its attention coefficients can be deceptively updated by the graph learning module. The nonlinear temporal relationships relating to temperature are characterized using a gated temporal convolutional module. The features between the sensors on the edge, which are essential, are further considered in the Graph Neural Network with Optimised Attention Mechanisms model (GNN-OAMs) [29], which was used to predict 3D ocean temperature. A random forest module was designed to capture non-stationary temporal dependencies, and dynamic spatial dependencies were modeled by combining multiple adjacency matrices. A neural network incorporating a spatial–temporal coupling mechanism was proposed in [30], in which a parallel convolution attention mechanism and a temporal sequence attention mechanism were employed to comprehensively extract key features in each module. Physics-informed neural networks (PINNs) [31] were applied for spatio-temporal temperature field prediction, where the effects of spatio-temporal variables were described by a dual thread convolutional network module. The authors of [32] designed a physics-informed deep learning model for multi-depth lake temperature prediction, consisting of two LSTM modules for spatial and temporal prediction, respectively, and one physical module for providing simulation data for training as well as ensuring consistency between model predictions of temperature and the physical mechanisms. The spatial and temporal co-evolution processes were taken into account in this model.

Although relationships between sensors have been studied for temperature prediction using graph networks, they are often characterized by graph embedding or graph encoders. Therefore, these relationships remain unclear. In this study, we aimed to develop an explicit graph adjacency matrix that considers the relationships between sensors based on their locations. Note that the impacts between sensors can be directional. For example, in a cold snap, neighboring areas with low temperatures can affect areas with high temperatures, causing the high-temperature areas to cool down. Therefore, a graph adjacency matrix was designed as a directed graph in order to present the directional relationships between sensors. Then, a directed-graph-based attention module was designed to extract the spatial relationships between temperature data over different sensors; the temporal relationships pertaining to the temperature data were extracted using a graph-gating module. Thereafter, the temperature around the sensors could be predicted based on the fusion of the directed-graph-based spatial–temporal features of the temperature data. The contributions of this manuscript are summarized as follows:

(1) A directed graph adjacency matrix was built based on the locations of temperature sensors, providing an explicit physics-guided module for modeling spatial and temporal relationships between temperature data. It can also describe the directional impacts between temperature data.
(2) A directed-graph-guided attention module and gating module were developed to capture the spatial and temporal relationships between temperature data across different sensors. Such physical informed features will greatly facilitate temperature prediction performance.
(3) A directed-graph-based fusion module was designed to integrate the spatial and temporal features for temperature prediction, demonstrating superior performance over real-world datasets collected in southern China.

## 2. Data Used and Methods

### 2.1. Study Area and Data Used

**Figure 1:**
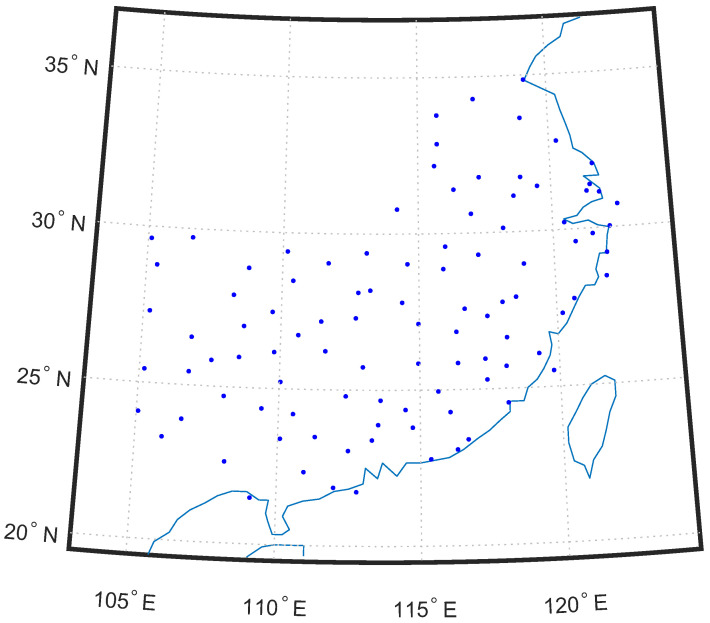
The locations of the 100 temperature-monitoring stations. A dot indicates a station.

In this paper, we study the temperature prediction problem in 100 cities in southern China [33]. The cities are shown in Figure 1 based on their latitudes and longitudes. We aimed to predict the temperature of one city based on a short period of its historical data and the relationships between the temperatures of its neighboring cities. Daily maximum temperatures over a period of 365 days from 1 January 2018 to 31 December 2018 of these 100 cities were collected from [34].

### 2.2. Problem Formulation

Temperature data are collected over monitoring stations, which are denoted by $x={\left(x_{0},x_{1},\ldots ,x_{N-1}\right)}^{T}$, with the temperature $x_{n}$ collected at the station $v_{n}$. *N* is the number of monitoring stations, and ${\left(\cdot \right)}^{T}$ denotes the transpose of the vector (·). We use $x^{k}$ to denote the temperature data in time instant *k*. In this paper, the temperature prediction problem is studied based on historical temperature data

(1) $$X^{\left(k\right)}=\left[x^{k-1},x^{k-2},\ldots ,x^{k-M}\right]$$

and the locations of the temperature-monitoring stations

(2) $$Y=[y_{1},y_{2},\ldots ,y_{N}].$$

$y_{i}$ is the location of the *i*-th station, and *M* is the length of time series to be utilized for temperature prediction. The temperature prediction model is formulated by

(3) $$\min∥x^{k}-f\left(X^{\left(k\right)},A\left(Y\right)\right)∥,$$

where A(Y)) is a physics-informed directed adjacency matrix to be designed based on the locations of the stations, and f(·) is the temperature prediction model to be designed.

### 2.3. Methods

**Figure 2:**
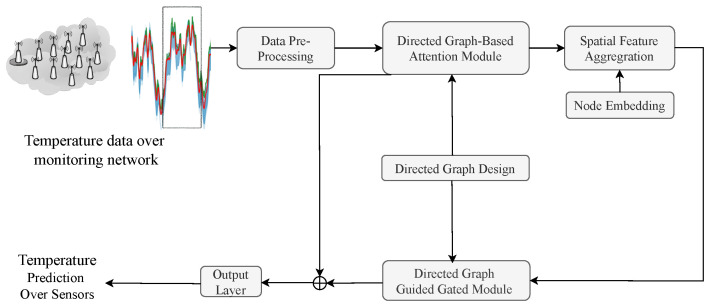
The framework of the proposed physics-informed directed-graph-based temperature prediction model.

This section presents the physics-informed directed-graph-based temperature prediction model. The framework of the proposed model is shown in Figure 2. The temperature data are pre-processed and then used to extract the directed-graph-based spatial and temporal features for temperature prediction. The directed graph design module is shown in Section 2.3.1, and the directed-graph-based attention module, which is used to model spatial features of temperature data over different stations, is shown in Section 2.3.2. The physics-informed gate module, used to extract temporal features of temperature data, is presented in Section 2.3.3. Finally, a graph-based fusion module used for temperature prediction is described in Section 2.3.4.

#### 2.3.1. Directed Graph Design Module

Two stations that are farther apart engage in weaker interactions, and two stations that are closer together engage in stronger interactions. Hence, the directed adjacency matrix was modeled according to the distance between two stations, which is defined by

(4) $$d_{i,j}=∥y_{i}-y_{j}∥$$

for 1 ≤ i,j ≤ N. Then, the adjacency matrix A can be defined by [33]

(5) ai,j=e−d˜i,j2/e−d˜i,j2∑k∈Nie−d˜i,k2∑l∈Nje−d˜j,l2∑k∈Nie−d˜i,k2∑l∈Nje−d˜j,l2,

where $a_{i,j}$ is the element at the *i*-th row and *j*-th column of the adjacency matrix A. The parameter ${\tilde{d}}_{i,j}$ is defined by

(6) $${\tilde{d}}_{i,j}=d_{i,j}/\sigma ,$$

where σ is a constant value. $N_{i}$ and $N_{j}$ denote the nearest-neighbor sensor set of the *i*-th station and the *j*-th station, respectively.

Note that the neighbor set of station $v_{i}$ and the neighbor set of station $v_{j}$ are different; i.e., $N_{i}\neq N_{j}$. Therefore, the adjacency matrix A defined by Equation (5) is a directed adjacency matrix, which can model the directional relations between temperature data collected at different stations. A long distance ${\tilde{d}}_{i,j}$ will lead to a small $a_{i,j}$ in A, indicating weak interaction between station $v_{i}$ and $v_{j}$, and vice versa. Note that the influence of temperatures at two stations is directed. For example, during a cold snap, stations with lower temperatures will affect neighboring stations with higher temperatures. Therefore, a directed asymmetric adjacency matrix is better suited to characterizing the influence of the temperatures at different stations in the prediction model.

#### 2.3.2. Directed-Graph-Based Attention Module

The computation of the feature of the input temperature is denoted as

(7) $${\tilde{x}}^{k}=Ax^{k}$$

a vector of size N×1. Then, the input feature is defined by

(8) $$s_{i}^{k}=C{\left[{\tilde{x}}_{i}^{k-1},{\tilde{x}}_{i}^{k-2},\ldots ,{\tilde{x}}_{i}^{k-M}\right]}^{T},$$

where C is a matrix of size R×M, ${\tilde{x}}_{i}^{k}$ is the *i*-th element of ${\tilde{x}}^{k}$, and $s_{i}^{k}$ is of size R×1. $a_{i,j}$ denotes the (i,j)-th element of matrix A and *b* denotes the scalar. Then, the attention coefficient can be computed by [35]

(9) $$\alpha_{i,j}=ba_{i,j}\frac{exp(\pi (i,j))}{\sum_{n\in N_{n}}exp\left(\pi \left(i,n\right)\right)},$$

where

(10) $$\pi \left(i,j\right)=LeakyReLU(a^{T}\left({\tilde{s}}_{i}^{k}⊕{\tilde{s}}_{j}^{k}\right)),$$

and

(11) $${\tilde{s}}_{i}^{k}=v_{i}⊕Hs_{i}^{k}.$$

H is a coefficient matrix of size L×R, $v_{n}$ is a node embedding of size d×1, and a is a column vector of size (d+L)×1.

The coefficient $a_{i,j}^{r}$ in Equation (9) guarantees a multiple hops fusion in computing attention coefficients. The aggregated spatial feature at node *n* can thereafter be computed by

(12) $${\hat{s}}_{n}^{k}=ReLU\left(\alpha_{n,n}Hs_{n}^{k}+\sum_{j\in N_{n}}\alpha_{n,j}Hs_{j}^{k}\right).$$

#### 2.3.3. Directed-Graph-Based Gate Module

The directed graph adjacency matrix is incorporated in modeling the temporal relationship between temperature data. This is denoted as

(13) $$g_{n}^{k}=s_{n}^{k}⊕{\hat{s}}_{n}^{k},$$

(14) $$q_{n}^{k}=\sigma \left(S_{q}g_{n}^{k}+U_{q}h_{n}^{k-1}+\sum_{j\in N_{n}}\alpha_{n,j}V_{q}h_{j}^{k-1}+d_{q}\right),$$

and

(15) $$p_{n}^{k}=\sigma \left(S_{p}g_{n}^{k}+U_{p}h_{n}^{k-1}+\sum_{j\in N_{n}}\alpha_{n,j}V_{p}h_{j}^{k-1}+d_{p}\right),$$

where $S_{q}$, $S_{p}$, $U_{q}$, $U_{p}$, $V_{q}$, and $V_{p}$ are coefficient matrices to be determined. $d_{q}$ and $d_{p}$ are column vectors, and σ is the Sigmoid function. Then, the temporal feature can be obtained by the following equation [36]

(16) $$h_{n}^{k}=\left(1-p_{n}^{k}\right)⊙h_{n}^{k-1}+p_{n}^{k}⊙{\tilde{h}}_{n}^{k},$$

where

(17) $${\tilde{h}}_{n}^{k}=\tanh\left(S_{h}g_{n}+U_{h}\left(p_{n}^{k}⊙h_{n}^{k-1}\right)+\sum_{j\in N_{n}}\alpha_{n,j}V_{h}h_{j}^{k-1}+d_{h}\right).$$

$S_{h}$, $U_{h}$, and $V_{h}$ are coefficient matrices to be determined. Hence, the temporal feature of temperature of station *n* at time instant *k*, i.e., $h_{n}^{k}$, can be recursively calculated based on the temporal feature at time instant k−1, i.e., $h_{n}^{k-1}$ based on (16).

#### 2.3.4. Physics-Informed Fusion Module

For $u_{n}^{k}$, the fusion feature is denoted as

(18) $$u_{n}^{k}=h_{n}^{k-1}⊕{\hat{s}}_{n}^{k}⊕{\tilde{x}}_{r}^{k}⊕w_{n},$$

where ${\tilde{x}}_{r}^{k}$ is the *r*-th column of ${\tilde{X}}^{\left(k\right)}$ defined in (7) and $w_{n}$ is the node embedding. The fusion feature integrates the temporal feature $h_{n}^{k-1}$, the spatial feature ${\hat{s}}_{n}^{k}$, the input temperature feature ${\tilde{x}}_{r}^{k}$, and the temperature embedding at station *n*. Thereafter, a multilayer perceptron model is designed to predict the temperature distribution; i.e.,

(19) $${\hat{x}}^{k}=MLP\left(u_{n}^{k}\right).$$

## 3. Results and Discussion

### 3.1. Numerical Experiments

**Figure 3:**
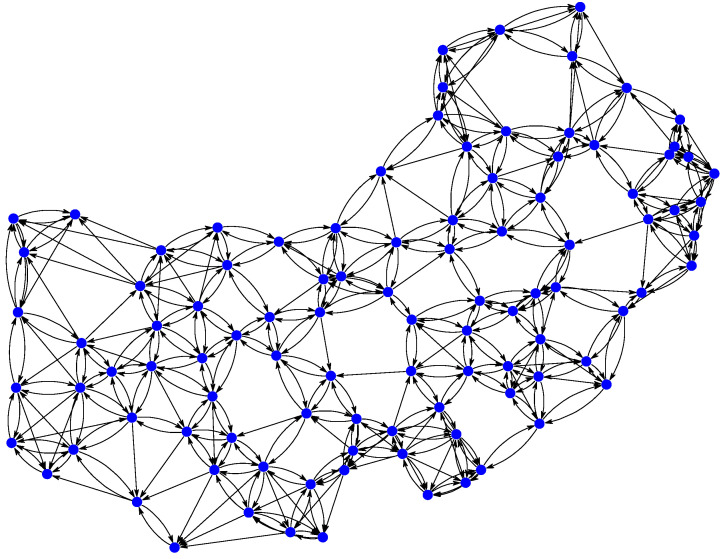
The directed graph of the 100 cities constructed based on the locations of temperature-monitoring stations. A dot indicates a station.

In this section, the proposed physics-informed directed-graph-based temperature prediction model is illustrated based on a real-world temperature dataset collected in 100 cities of China [33,34]. Daily temperatures over a period of 365 days were studied, where 100 days of temperature data were used for training, 85 days of data were used for validation, and 180 days of data were used for testing. The directed adjacency matrix was computed using Equation (6), where the distance between two temperature-monitoring stations is computed based on their latitudes and longitudes and the coefficient σ=200. Each station chooses the five closest stations to construct a directed adjacency matrix A. The directed graph is shown in Figure 3. A directed edge between two cities indicates that the temperature of the arc-tail city is considered in forecasting the temperature of the arc-head city. However, the temperature of the head city does not take into account the prediction of temperature of the tail city. If there is no directed edge between two cities, the temperature relationships between these two cities are not considered when forecasting the temperature. The configuration of the experimental parameters is summarized in Table 1.

**CST-GL model:** The correlation-aware spatial–temporal graph learning model [37] is considered as the graph neural network baseline model. In CST-GL, the graph adjacency is constructed by correlations of temperatures at different stations. Hence, the graph adjacency matrix of CST-GL is a symmetric matrix. The spatial features of CST-GL are modeled by a conventional graph convolution module and the temporal features of temperatures are modeled by a convolution module.

**Figure 4:**
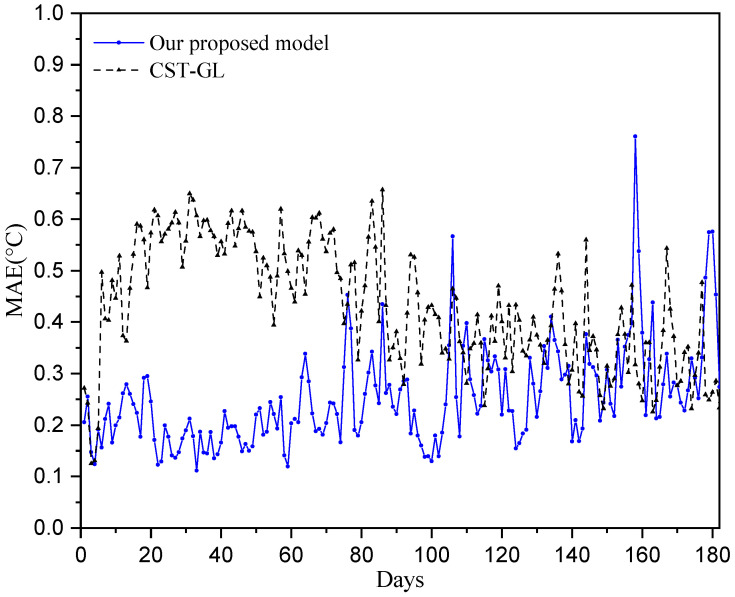
Prediction performance of daily temperatures over 180 testing days.

We use the mean absolute error (MAE) to evaluate the daily prediction performance [37], i.e.,

(20) $$MAE_{daily}=1/N∥{\hat{x}}^{k}-x^{k}∥,$$

where ${\hat{x}}^{k}$ is the predicted temperature and $x^{k}$ is the true temperature collected from stations. The prediction errors over all 100 stations are taken into account, and the daily prediction performance is shown in Figure 4. It can be seen that the mean absolute error for each sensor is less than 0.7 °C, and more than half of the temperature prediction errors are less than 0.3 °C, while most of the MAE of CST-GL are bigger than 0.3 °C, which demonstrate the effectiveness of the proposed physics-informed directed-graph-based temperature prediction model.

To further investigate the temperature prediction performance, the mean absolute errors of each sensor over all the 180 days are considered. ${\hat{x}}_{n}$ and $x_{n}$ denote the temperature prediction value for station *n* over all 180 days and the true temperature collected by stations, i.e.,

(21) $${\hat{x}}_{n}=\left[{\hat{x}}_{n}^{1},{\hat{x}}_{n}^{2},\ldots ,{\hat{x}}_{n}^{T}\right].$$

and

(22) $$x_{n}=\left[x_{n}^{1},x_{n}^{2},\ldots ,x_{n}^{T}\right].$$

**Figure 5:**
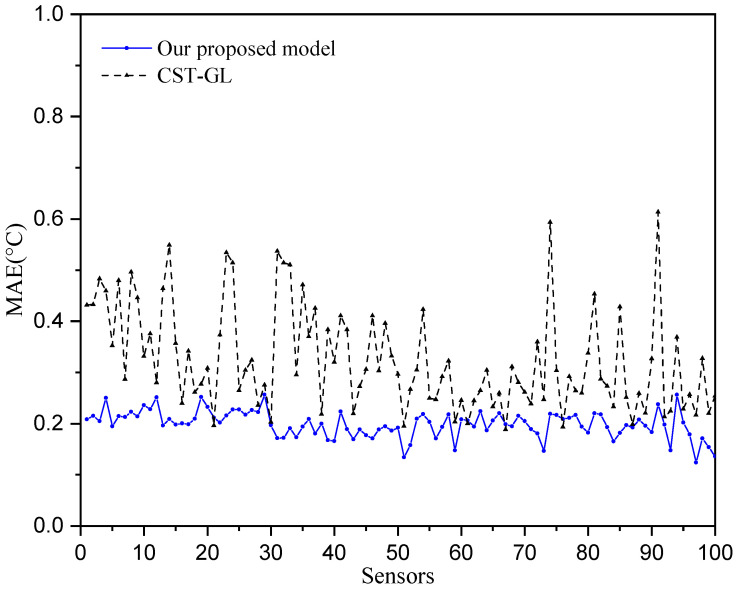
Temperature prediction performance of all 100 sensors.

Then, the mean absolute error of sensors can be computed by

(23) $$MAE_{sensor}=1/T∥{\hat{x}}_{n}-x_{n}∥,$$

where T=180. The mean absolute errors of each sensor are shown in Figure 5, where we can see that all of the mean temperature prediction error are smaller than 0.25 °C, while most of the MAE of the CST-GL model are greater than 0.3 °C. This demonstrates the superior prediction performance of the proposed model.

**Figure 6:**
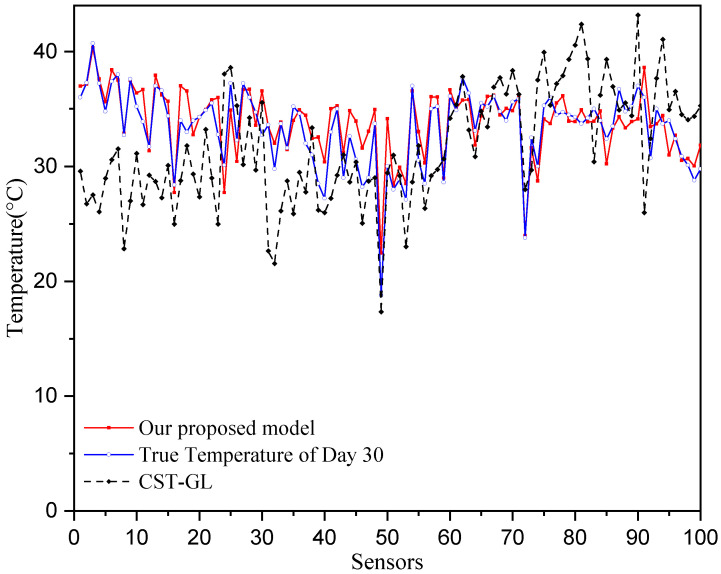
Comparison of the true temperatures and the predicted temperatures of all 100 sensors of Day 30.

**Figure 7:**
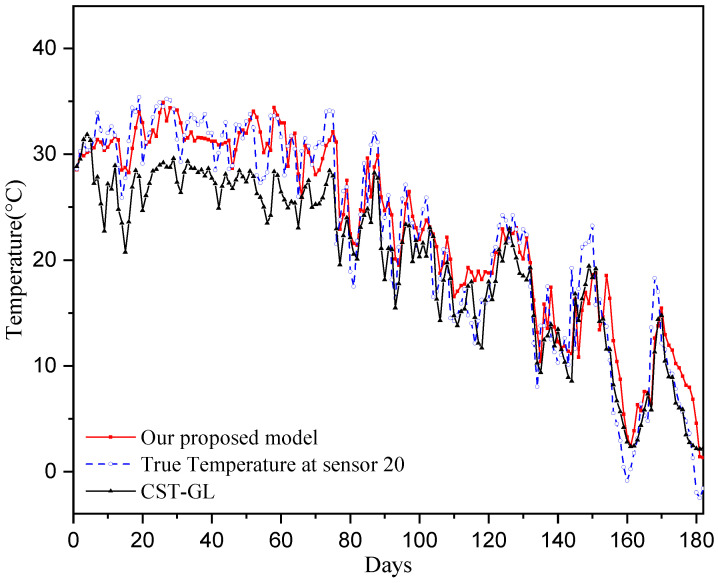
Comparison of the true temperatures and the predicted temperatures of sensor 20 over the 180 testing days.

To provide straightforward comparisons between true temperatures and predicted temperatures, we show the comparisons on Day 30 across all sensors in Figure 6 and show the comparisons at sensor 20 for the period of 180 days in Figure 7. It can be seen that the prediction temperatures of our physics-informed directed-graph-based model are very close to the true values. The temperature prediction of the CST-GL model show greater variations around the true temperature than those of our proposed model. Therefore, the proposed physics-informed directed-graph-based model can capture the spatial and temporal relations between temperature data collected at different stations. This is achieved using the graph attention module and the graph-based gate module, which are developed on the directed graph adjacency matrix. These modules significantly improve temperature prediction performance.

### 3.2. Discussions

Figure 4, Figure 5, Figure 6 and Figure 7 show that the proposed physics-informed directed-graph-based temperature prediction model demonstrates much better prediction performance than the CST-GL model. In CST-GL, graph adjacency is constructed using correlations of temperatures at different stations. Hence, CST-GL is a data-driven model. Moreover, the graph adjacency matrix of CST-GL is a symmetric matrix, which may not accurately characterize the relationships between temperatures at different stations because the influence of the temperatures at the two stations may be different. Therefore, the advantages of our proposed temperature prediction model is evident in the directed graph adjacency matrix constructed using the locations of stations. The spatial features of CST-GL are modeled by a conventional graph convolution module and the temporal features of temperatures are modeled by a convolution module. In our proposed temperature prediction model, spatial features are integrated with a physics-informed directed graph adjacency matrix. Temporal features are characterized using a directed-graph-based gate module, which is able to explicitly model the relationships between temperatures at time instant *k* and previous time instants.

**Figure 8:**
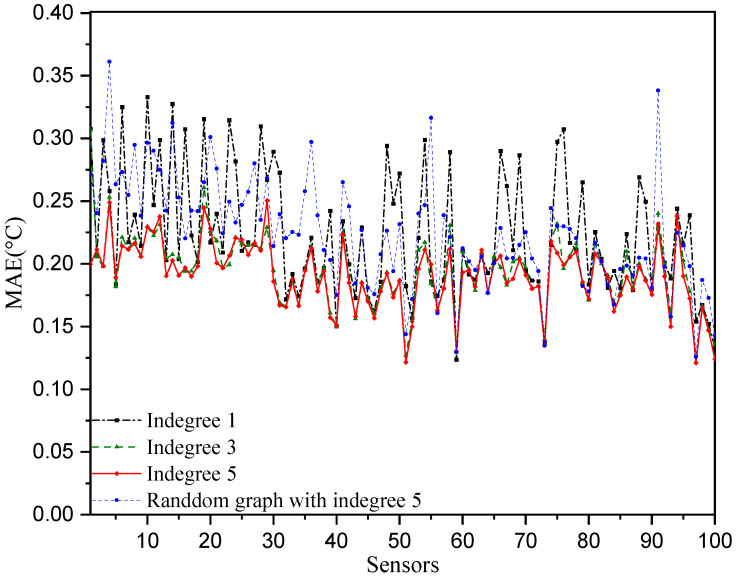
Temperature prediction performance with different directed graph adjacency matrices.

**Figure 9:**
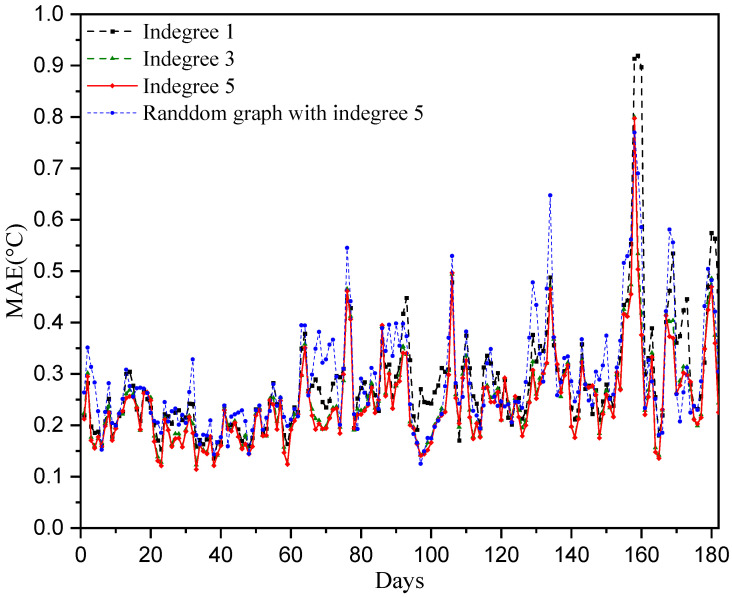
Prediction performance of daily temperatures over 180 testing days with different directed graph adjacency matrices.

In the aforementioned numerical experiments, each station selects its five closest neighboring stations for temperature prediction. Hence, the in-degree of each node of the constructed directed graph adjacency matrix is five. To investigate the influence of the directed adjacency matrix on the temperature prediction performance, the adjacency matrices of in-degree 1 and 3 are considered. That is, each station selects the closest one (or the closest three) neighboring stations for temperature prediction. The results are shown in Figure 8 and Figure 9. We can observe that the directed adjacency matrix with in-degree of 5 has the best temperature prediction performance across the sensors as well as over the 180 test days. The performance is significantly degraded when only the closest station is selected in the temperature prediction. Furthermore, we consider a random adjacency matrix with an in-degree of five in the prediction model. That is, each station randomly selects five other stations for temperature prediction. Its performance is worse than the case of the adjacency matrix with the closest five neighboring stations. The reason for this may be that the relationships regarding temperature at randomly selected stations are not as strong as those at the five closest neighboring stations.

**Figure 10:**
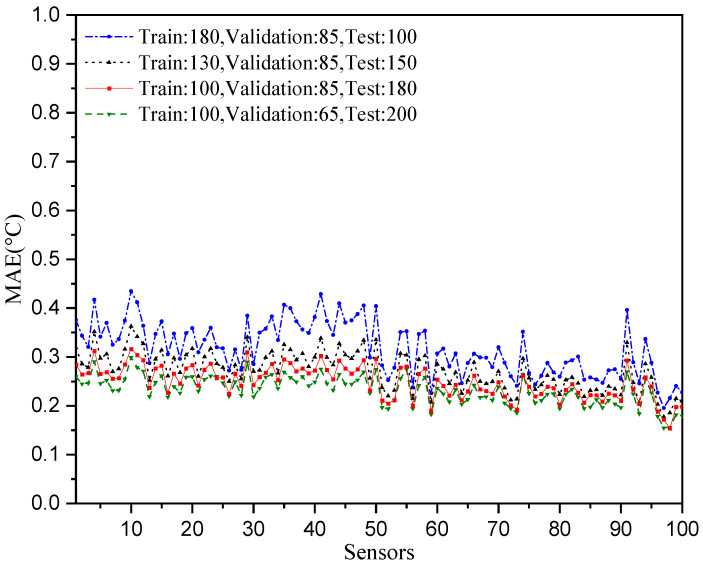
Prediction performances with different data length configurations.

The temperature prediction performances with different data length configurations are shown in Figure 10. We can observe that both the number of train data and test data cannot be too small. A small number of training data cannot provide enough data with which to learn the relationships between the temperatures, and a greater number of test data will make the mean absolute error of prediction performance more robust.

## 4. Conclusions

In this paper, we propose a physics-informed directed-graph-based temperature prediction model, which consists of a directed graph design module, a directed-graph-based attention module, a graph-based gating module, and a fusion module with spatial and temporal features for temperature prediction. The directed graph adjacency matrix was constructed based on the locations of different temperature-monitoring stations; it has a physics-based graph structure to characterize the relationships between temperature data and is capable of modeling an asymmetric relationship. The directed adjacency matrix is explicitly incorporated in the graph attention module, guaranteeing a multiple-hop attention mechanism. The directed adjacency matrix is introduced in the graph-based gating module to capture the temporal features of temperature, and the fusion model is designed to integrate the spatial, temporal, and directed graph structure to improve temperature prediction performance. The numerical experiments demonstrate that the proposed model can provide temperature prediction performance superior to that of the baseline model.

## Figures and Tables

**Table 1:** Experimental configuration.

Parameters	Value
Slide Window Length *M*	5
Number of Cities *N*	100
Embedding Size *d*	128
Feature Length *L*	256
Feature Length *R*	128
Learning Rate	4 × $10^{-5}$
Number of Epochs	400
Weight-Decay Parameters	0.0001
Batch Size	32
σ	200

## Data Availability

Dataset available on request from the authors.
